# Immune-Mediated Cutaneous Paraneoplastic Syndromes Associated With Hematologic Malignancies: Skin as a Mirror of Hematologic Neoplasms

**DOI:** 10.7759/cureus.19538

**Published:** 2021-11-13

**Authors:** Jose C Alvarez-Payares, Angel Molina, Simon Gallo, Julian Ramirez, Juan Hernandez, Fernando Lopez, Sara I Ramirez-Urrea, Cristian Álvarez

**Affiliations:** 1 Internal Medicine, Universidad de Antioquia, Medellin, COL; 2 Dermatology, Universidad de Antioquia, Medellin, COL; 3 General Medicine, Fundación Universitaria San Martín, Medellin, COL; 4 Internal Medicine, University of Sucre, Sincelejo, COL

**Keywords:** neutrophilic dermatosis, cutaneous manifestations of systemic disease, paraneoplastic syndrome, hematologic malignancies, hematology disorders

## Abstract

Malignant neoplasms may present as paraneoplastic syndromes with mucocutaneous manifestations, which may or may not be chronologically associated. The pathophysiological mechanism is complex and not completely understood; therefore, definitive diagnosis may be achieved with a precise differential diagnosis based on the morphology of skin lesions, clinical picture, and histological pattern. The complexities, and low frequency, make the therapeutic approach quite challenging; consequently, the cornerstone of therapy is the eradication of the underlying neoplasms. Corticosteroids are the therapy of choice for most of these immune-mediated manifestations, but for the most part, the successful resolution requires the eradication of the underlying malignancy.

## Introduction and background

Hematologic malignancies (HMs) represent a heterogeneous group of neoplastic disorders. According to cellular lineage, these are divided into myeloid and lymphoid neoplasms. Paraneoplastic syndromes may emerge before, during, or after the underlying neoplasm is detected, and its course may or may not be related [1].

A paraneoplastic syndrome consists of a process when an underlying neoplasm has a causal relationship with a group of clinical manifestations - aside from the tumor and its metastases [2]. Due to multiple organs being involved and an extensive variety of neoplasms associated (both solid and hematologic), a wide range of these syndromes have been described [3].

Even though no certain data exist about the pathogenesis of these syndromes, two premises have risen. These include the secretion of functional proteins that trigger a remote response, and the induction of antibody production, which cross-react with other tissues [4].

In the past few years, cutaneous manifestations have become more frequent in HMs and up to 25% are due to paraneoplastic cutaneous syndromes, and of these, up to half are immune-mediated. In a descriptive study, the most common HM with cutaneous manifestations was leukemia (38.3%), followed by non-Hodgkin's lymphoma (26.7%) and multiple myeloma (21.7%) [3].

Dermatological manifestations associated with HM can be divided into specific and non-specific. Specific manifestations include massive skin infiltration of neoplastic cells, which is known as leukemia cutis [5]. Non-specific manifestations include dermatologic changes due to bone marrow insufficiency (pallor and ecchymosis), skin infections, adverse reactions to oncologic treatment, as well as immune-mediated diseases that will be discussed in this review. Furthermore, epidemiological and clinical aspects are emphasized, presenting the skin as an important organ for the diagnosis of this type of malignancy.

## Review

Pathophysiology

The risk of developing an immune-mediated dermatosis associated with an HM depends on many factors, including genetic and epigenetic ones, the subtype of HM, and the consequences of specific treatment [3]. Central tolerance, regulated by negative selection and peripheral T cells, is affected in patients with HM. Thymoma, or lymphoproliferative entities, may assist the escape of autoreactive T cells, which could therefore trigger disease reactions, such as paraneoplastic pemphigus (PP) [6].

Autoreactive peripheral cells may become anergic if no antigen-presenting cell is found. However, tumor cells, such as Hodgkin's lymphoma (HL) and B-cell chronic lymphocytic leukemia (CLL), may express molecules such as β2 microglobulin, or major histocompatibility complex type I and II, which can favor the expansion and differentiation of the autoreactive cells [7]. Neoplastic cells recruit regulatory T cells, which produce interleukin-10 to avoid immune surveillance, a situation that does not avoid immune reactions, as these cells also produce interleukin-4 and interleukin-17, which promote tumor tolerance and peripheral inflammation [6,7].

HM has been associated with an imbalance among T cells and cytokine production. An example is a shift to a T helper 2 (Th2) response, and the increase of Th2/T helper 1 (Th1) relationship, in diseases such as HL, acute myeloid leukemia (AML), and chronic myeloid leukemia (CML) [8]. This situation may be responsible for different phenomena such as neutrophil cutaneous recruitment or the stimulation of autoantibody production. Furthermore, an increase of interleukin-1 and interleukin-6 is observed in CML, which could be related to neutrophilic dermatosis pathogenesis [9]. Additionally, in HL and B-cell CLL, a microenvironment enriched with Th17 has been observed, which is thought to be related to the pathogenesis of neutrophilic and autoimmune blistering dermatoses. Mechanisms implied on immune-mediated skin disorders’ pathogenesis in patients with hematologic malignancies are described in Table 1.

**Table 1:** Mechanisms implied on immune-mediated skin disorders’ pathogenesis in patients with hematologic malignancies. Th = T helper cell; AML = acute myeloid leukemia; CML = chronic myeloid leukemia; HL = Hodgkin’s lymphoma; NHL = non-Hodgkin’s lymphoma; CLL = chronic lymphocytic leukemia.

Hematologic malignancy	Mechanism
Lymphoid lineage malignancies	Central T cell tolerance disorder (immature and autoreactive T cell escape from thymus negative selection)
Lymphoid and myeloid lineage malignancies	Peripheral T cell tolerance disorder (co-stimulating signals for autoreactive T cells by neoplastic cells)
B-cell lymphoproliferative disorders	Autoantibody production by neoplastic cells
CML, AML, HL, CLL, and B-cell cutaneous lymphoma	Th1/Th2 cell imbalance
HL, NHL, acute leukemia, and CLL	Th17 cell activity increase
Lymphoid and myeloid lineage malignancies	Cytokine imbalance

Immune-mediated cutaneous paraneoplastic syndromes in HM

The immune-mediated cutaneous paraneoplastic syndromes in HM that will be mentioned during this review are (1) neutrophilic dermatoses (ND), (2) eosinophilic dermatoses (ED), (3) blistering dermatoses, and (4) miscellaneous (vasculitis, connective tissue diseases, and granulomatous dermatoses).

Neutrophilic Dermatoses

ND is a heterogeneous group of clinically polymorphic dermatoses characterized by mature neutrophil infiltrates and no evidence of infection [10]. The most frequent ones are Sweet's syndrome, pyoderma gangrenosum (PG), erythema elevatum diutinum (EED), subcorneal pustular dermatosis, and neutrophilic eccrine hidradenitis (NEH) [1,2,11]. Evidence has been found that in these dermatoses, neutrophils may be clonally related to neoplastic cells and may have been differentiated from them [12]. If such a hypothesis is confirmed, these entities could no longer be considered paraneoplastic syndromes.

Sweet's syndrome: About a fifth of the patients diagnosed with Sweet's syndrome suffer from an associated malignancy with a majority (85%) of them related to HM [13], and have a prevalence of AML and myelodysplastic syndrome (MDS) that oscillates between 15% and 27% [14]. It has been well described in CML, B-cell CLL, monoclonal gammopathies (mainly immunoglobulin G [IgG]), and multiple myeloma (MM), and less frequently in HL, non-Hodgkin's lymphoma (NHL), and myeloproliferative disease (MPD) [15]. The mortality rate seen in patients with Sweet's syndrome and associated HM is higher (44%) when compared to 14% in those without HM [15,16].

**Figure 1:**
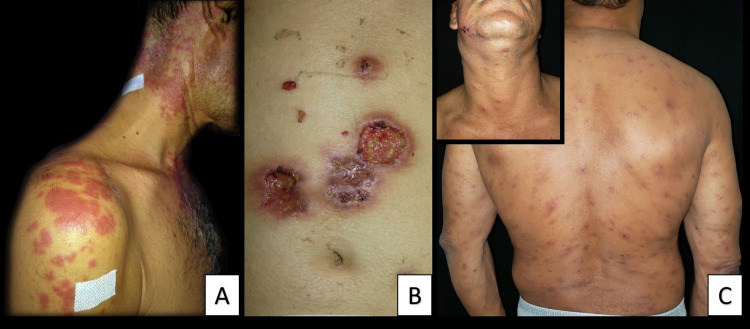
A. Sweet's syndrome. Infiltrative erythematous and well-defined plaques on the neck, chest, and shoulder in a patient with hairy cell leukemia. B. Pyoderma gangrenosum. Ulcers with bluish, overhanging borders in the abdomen. A central lesion with cribriform appearance. C. Hematologic malignancy-associated eosinophilic dermatosis. Erythematous plaques and papules with generalized vesicles in a patient with mantle cell lymphoma - note cervical lymphadenopathy. Pictures courtesy of the University of Antioquia Dermatology Service.

Clinical presentation of Sweet's syndrome can be easily mistaken as an infection, as it presents with fever, neutrophilia, nodules, erythematous, and/or painful plaques, which may be associated with pseudo vesiculation or pustules, besides blistering and unusual subcutaneous forms [17]. Usually located in the upper limbs, torso, back, head, and neck (Figure 1A), plaques do not respond to antibiotics generally [14]. Furthermore, patients may also present with extracutaneous manifestations that include conjunctivitis, episcleritis, arthritis, myalgias, glomerulonephritis, hepatitis, neutrophilic alveolitis, encephalitis, myocarditis, splenomegaly, among others [15-17]. Cutaneous manifestations usually occur months or years before HM diagnosis (although they can appear simultaneously), and they may recur after clinical remission, during HM relapses [17].

Pyoderma gangrenosum: PG is an uncommon inflammatory disease [18]. In a systematic review with 823 PG cases conducted by DeFilippis et al., it was found that inflammatory bowel disease, polyarthritis, and HM were associated with PG in 65.2%, 16.1%, and 12.5% of the cases, respectively [19]. Of the cases associated with HM, 25% corresponded with MDS, 22% with monoclonal gammopathy of uncertain significance (MGUS), 12% with AML, and 6.4% with MM [20]. The most common types of PG associated with HM are ulcerative and blistering [18], especially in myeloproliferative syndromes. IgG gammopathies are more frequent; however, the ones associated with PG are immunoglobulin A (IgA) [16]. The association between HM and PG has been observed as a cause of death [16,21], mainly in AML, CML, MDS, MM, MGUS, and lymphoma.

Lesions evolve from a papule or inflammatory pustule to a violaceous, extremely painful ulcer, with elevated borders, sterile purulent exudate, and a necrotic base. Other subtypes have been described, such as blistering, pustular, vegetative, and extracutaneous [18]. The affected zones usually are lower limbs, specifically the anterior tibial surface (Figure 1B) [18].

Erythema elevatum diutinum: EED is a chronic, localized form of leukocytoclastic vasculitis [22] and usually precedes HM for years [16]. It has been reported with manifestations of a variety of HM, primarily monoclonal gammopathy (of IgA isotype), lymphomas, and MDS [22-24]. EED is rare, with approximately 250 cases reported in the literature until 2011 [22]. It typically presents as papules and erythematous nodules, with a red/violet color, localized on extensor surfaces [16,22-24].

Subcorneal pustular dermatosis: Also known as Sneddon-Wilkinson disease, it is a rare, relapsing, chronic pustular eruption, characterized by flaccid subcorneal pustules that contain neutrophils in histopathology [25]. The least associated HM is IgA myeloma, aplastic anemia, lymphomas, and CLL [16]. Cutaneous signs may appear years before HM diagnosis, and they may improve with the treatment of the underlying malignancy [16,25,26].

Neutrophilic eccrine hidradenitis: It is a rare dermatosis, characterized by infiltrated papules or plaques like in Sweet's syndrome [16], with neutrophil infiltrates surrounding eccrine glandules. These lesions may be asymptomatic, itchy, or painful, and are usually located in the trunk, face, or limbs. Patients usually present with a self-resolving course [16]. Bachmeyer et al. conducted a literature review of 51 cases of NEH that indicated that 67% had AML [27]. Other HMs associated were B-cell CLL, CML, HL, and NHL [27]. Most patients (84%) received chemotherapy (specifically cytarabine and anthracyclines) before NEH started. Generally, NEH patients present with a spontaneously resolving course in a few days or weeks, and no specific treatment is required [16]. In most cases, it is considered an adverse effect of chemotherapy.

Eosinophilic Dermatoses

ED is a heterogeneous group of cutaneous eruptions that predominantly occur in patients with indolent lymphoproliferative disorders, mainly CLL, in up to 6-8% of these cases [28]. Some patients, mainly those with acute leukemia, chronic myeloproliferative disorders, and plasma cell dyscrasias, may also present this dermatosis [29]. It usually presents months or years after HM diagnosis [30]. Nonetheless, a minority of patients can also present with the cutaneous rash up to 10 years before HM diagnosis [16].

These patients may develop "insect bite-like" reactions, due to T cell proliferation and the release of interleukin-5 [31]. Some controversy surrounds this dermatosis and its denomination as a paraneoplastic syndrome, given some findings of tumor invasion in the skin [16]; hence, two hypotheses have arisen: (1) that tumor cells infiltrate the skin and promote eosinophil activation; or (2) neoplastic treatment may trigger a persistent deviation of T cells to a Th2 phenotype, which may favor T cell recruitment on the skin and eosinophil activation in response to environmental triggers [16].

At least three main clinical patterns have been described [16]: (1) a blistering pattern, similar to bullous pemphigoid; (2) an “insect bite-like” pattern, characterized by discrete urticaria papules, sometimes with abundant vesicles similar to papular urticaria (Figure 1C); and (3) a cellulitis-like pattern, characterized by diffuse erythematous plaques or nodules similar to Wells syndrome. Patients may present with intense itching, which is often intolerable.

Eosinophilic dermatosis (ED) must be differentiated from T cell papulosis associated with B-cell neoplasms; these are characterized by itchy papules, vesicles, plaques, and nodules [32]. Its histopathology shows prominent T cell infiltrates, like cutaneous T cell lymphoma, specifically, folliculotropic mycosis fungoides. Just like ED, the rash presents in a relapsing manner despite treatment.

Blistering Dermatoses

**Figure 2:**
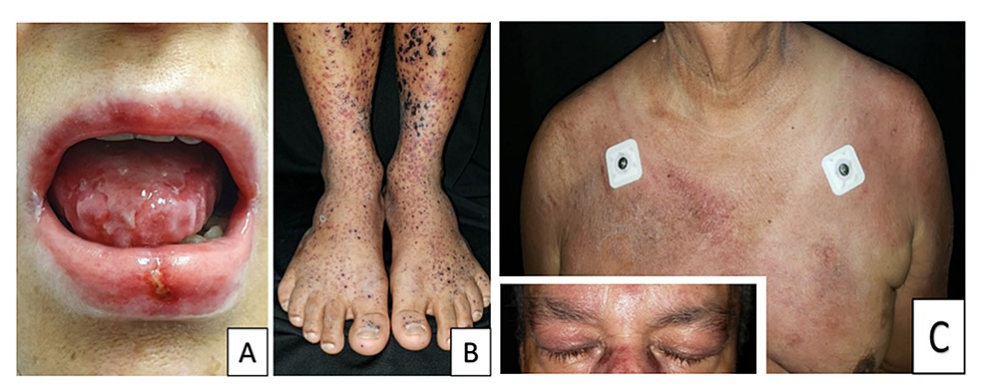
A. Paraneoplastic pemphigus. Oral mucositis with diffuse erosion areas involving lip and tongue mucosa in a patient with thymoma. B. Small vessel vasculitis. Palpable purpura with necrotic crusts in both lower limbs. C. Dermatomyositis. Heliotrope erythema and macules in trunk and shoulder (shawl sign). Pictures courtesy of the University of Antioquia Dermatology Service.

These are characterized by suprabasal acantholysis due to autoantibodies, principally IgG, directed against keratinocytes’ intercellular adhesive proteins [33]. Even though a higher prevalence and a possible association of malignancies with pemphigus vulgaris, pemphigus foliaceus, and bullous pemphigoid, the classic paraneoplastic entity is the PP - a rare, devastating autoimmune disease that affects skin and mucosa, presenting in patients with underlying malignancies, although it has been sporadically described even in the absence of tumors [16]. PP occurs in association with a variety of neoplastic disorders, the most frequent are HM-like lymphomas (45%), Castleman’s disease (15%), and leukemia (7%) [34]. Clinically, it differs from other forms of pemphigus by a more severe mucous involvement (Figure 2A) and greater resistance to immunosuppressant treatment, and the skin may present with flaccid or tense bullae, with an atypical target pattern, simulating erythema multiforme or Stevens-Johnson syndrome [16].

Miscellaneous

Plenty of paraneoplastic syndromes with skin involvement have been reported as a result of vascular disturbances, connective tissue abnormalities, or granulomatous infiltration [16].

Vasculitis: 5% of cutaneous vasculitides are associated with an underlying malignancy [35], which are HMs in 90% of cases, primarily chronic myelomonocytic leukemia, NHL, HL, CLL, and MM.

Paraneoplastic cutaneous vasculitis has a similar clinical course to idiopathic ones, i.e. palpable purpura, erythematous nodules, chronic and painful ulcers, livedo reticularis or racemosa, gangrene, and acral necrosis with a predilection for lower limbs (Figure 2B). Those with a paraneoplastic origin have a longer duration and present lower response rates to glucocorticoid/immunosuppressive treatment [16,36].

It may represent a diagnostic challenge to distinguish it from leukemic vasculitis, which is a rare presentation of leukemia cutis. This distinction is critical, as leukemic vasculitis is associated with poor prognosis, which correlates with the aggressive biological behavior of the underlying hematologic malignancy [16].

Connective tissue diseases: These diseases, such as systemic lupus erythematosus (SLE), Sjögren’s syndrome, and systemic sclerosis, all have been linked to a higher risk of HM, primarily B-cell lymphoma [16]. Marginal zone lymphomas and mucosa-associated lymphoid tissue (MALT) lymphomas are related principally to Sjögren’s syndrome, with an incidence 44 times higher than the general population [37].

Malignancies occur in 13-42% of patients with dermatomyositis, with a higher risk in the first two years after diagnosis. Paraneoplastic dermatomyositis is more common in solid tumors, although classic and amyopathic dermatomyositis has been reported in patients with HM (NHL, HL, AML, and MDS) [16]. HM-associated dermatomyositis carries a poor prognosis, with survival rates of 96.9%, 78.1%, and 51.4% at one, three, and five years, respectively [38].

Clinical features include the classic erythematous dermic papules on top of interphalangeal joints (known as Gottron papules), heliotrope rash, erythematous/poikilodermatous macules with a signature distribution (V sign, shawl sign, and holster sign), and nailfold telangiectasias (Figure 2C). The presence of erythroderma in dermatomyositis is a hallmark of malignancy, specifically of lymphoid source [39].

Granulomatous dermatoses: Clinical entities associated with paraneoplastic syndromes are annular granuloma (AG), cutaneous sarcoidosis, and palisaded neutrophilic granulomatous dermatitis, which have all been associated with a wide range of malignancies, such as HL, NHL, MDS, and CML [40]. Sarcoidosis patients have a five times higher risk of developing a lymphoproliferative disorder (mainly HL) [16]. Regarding the association between AG and lymphoma, AG may precede the lymphoma diagnosis, which occurs subsequently, with times ranging between five and 27 years [41]. Lymphomas are responsible for 56% of malignancy-associated AG, being HL the most common one.

HM-associated paraneoplastic dermatoses diagnosis

The cornerstone of a paraneoplastic syndrome diagnosis is based on the integration of history and physical examination findings. The skin lesion plays a fundamental role to guide any clinical suspicion, which is either ruled in or out according to histopathology. Nonetheless, it is important to highlight that the diagnosis of certain paraneoplastic syndromes is not limited to histologic studies, as these may require additional procedures, such as specific biochemical studies.

After the dermatosis has been identified, it is necessary to look for possible underlying malignancies. The diagnosis of paraneoplastic syndrome with skin manifestations and the diagnostic workup of underlying malignancy are described in Table 2.

**Table 2:** Clinical and laboratory characteristics of mucocutaneous paraneoplastic syndromes associated with hematologic malignancies. CBC: complete blood count; APR: acute phase reactants; HM: hematologic malignancies; HIV: human immunodeficiency virus; DM: dermatomyositis; CPK: creatine phosphokinase; AST: aspartate aminotransferase; ALT: alanine aminotransferase; LDH: lactate dehydrogenase; DIF: direct immunofluorescence; MDS: myelodysplastic syndrome.

Paraneoplastic syndrome	Clinical characteristics	Diagnostic tests	Histologic findings	Commentary
Neutrophilic dermatoses	Erythematous, painful, and edematous plaques or nodules, associated with sudden fever and neutrophilia	Complete blood count (CBC), acute phase reactants (APR), and skin biopsy. Consider other studies.	Edema, dermis neutrophilic infiltrate, absence of vasculitis.	The main differential diagnosis is infection. Sweet’s syndrome presents a rapid improvement with corticosteroid use.
Sweet's syndrome				
Pyoderma gangrenosum	Violaceous irregular ulcer with undermined borders, pus, and intense pain	Skin biopsy for pathologic and microbiologic study. Consider other studies.	Dense neutrophilic infiltrate, necrosis, and hemorrhage. Leukocytoclastic vasculitis may be observed.	It may be associated with autoinflammatory or autoimmune diseases more than HM.
Erythema elevatum diutinum	Erythematous or brown solid nodules and plaques on extensor surfaces, particularly joints. It may be associated with ophthalmopathy or arthralgias	Skin biopsy. Consider other studies, including the HIV test.	Leukocytoclastic vasculitis in the middle and superficial dermis; late lesions are associated with dermis fibrosis.	It may precede for years following hematologic malignancy.
Subcorneal pustular dermatosis	Annular grouping of pustules in trunk and skin folds	Skin biopsy. Consider other studies.	Subcorneal pustules filled with neutrophils. Secondary acantholysis may occur. Mixed superficial perivascular skin infiltrates.	Recurrent course. The main differential diagnosis is pustular psoriasis; however, history, histology, and dapsone response may help to differentiate them.
Eccrine neutrophilic hidradenitis	Erythematous/purpuric nodules, plaques, macules, or papules, which may be painful, located in the head, neck, or trunk	Skin biopsy	Neutrophilic infiltrate surrounding eccrine sweat glands.	May be associated with fever and neutropenia. Most frequently associated with chemotherapy but has been reported as a paraneoplastic phenomenon.
Eosinophilic dermatoses associated with hematologic malignancy	Polymorphic lesions, from papules, plaques, erythematous, urticarial-like nodules, vesicles, and blisters.	Skin biopsy	Interstitial infiltrate consists of lymphocytes, histiocytes, and plenty of eosinophils. Panniculitis or eosinophilic spongiosis and flame-like figures may be observed.	An adult disease. Hematologic malignancy diagnosis may precede, occur simultaneously, or after the skin condition.
T cell papulosis associated with B-cell neoplasms	Papules, vesicles, plaques, and recurring, itchy nodules predominantly in the head and neck	Skin biopsy	Dense T cell skin infiltrates, with some eosinophils, with perivascular and periadnexal disposition, suggesting folliculotropic mycosis fungoides. Neoplastic B cells may be identified.	Described in 2018 with 38 cases of skin eruptions in patients with B cell lymphoproliferative disorders [32].
Connective tissue diseases (dermatomyositis [DM])	Heliotrope erythema, Gottron papules and sign, poikiloderma plaques in trunk and thighs, and periungual telangiectasia	Skin biopsy, CPK, aldolase, AST, ALT, LDH, electromyography, and magnetic resonance imaging. Consider other studies according to clinical presentation.	Dermis mucin deposits, lymphocytic infiltrate, epidermal atrophy, vacuolar changes of the basal layer, and telangiectasis.	DM may be previous (40%), concomitant (26%), or following HM malignancy (34%) diagnosis [42]. Lymphoma risk is higher during the first year after the diagnosis.
Granulomatous dermatoses (annular granuloma)	Different clinical presentations: localized, generalized, subcutaneous, and perforans. Usually with papules or erythematous plaques with an annular or arciform distribution with the hypopigmented center.	Skin biopsy	Necrobiotic degeneration of connective tissue, palisading histiocytes. Hypercellular dermis with histiocytes infiltrating collagen fibers. Perivascular lymphocytic infiltrates and mucin deposits.	Multiple triggers and systemic diseases have been associated. When HM has been associated, it may precede the diagnosis for one to two years.
Blistering dermatoses	Painful erosions, hemorrhagic crusts, mainly in the oral mucosa. Polymorphic cutaneous eruption, flaccid or tense blisters, and lichenoid lesions	Skin biopsy for pathology and direct immunofluorescence (DIF). Consider other studies looking for an underlying malignancy.	Suprabasal acantholysis; basal vacuolization with lichenoid lymphocytic infiltrate. DIF shows intercellular or linear C3 or IgG deposits in the dermo-epidermal junction.	Clinical characteristics may distinguish paraneoplastic pemphigus from another pemphigus: severe oral mucosa involvement and lesion polymorphism [43].
Paraneoplastic pemphigus				
Miscellaneous (vasculitis)	Palpable purpura, inflammatory nodules, ulcers, acral necrosis, and livedo racemosa	Skin biopsy. Consider other studies.	Leukocytoclasia, perivascular infiltrates, erythrocyte extravasation, and vessel wall fibrinoid necrosis.	Polyarteritis nodosa has been reported in MDS and hairy cell leukemia [44].

Treatment

Steroids play a fundamental role in the treatment of immune-mediated dermatoses - both in idiopathic and paraneoplastic ones. However, there exists a high rate of recurrence in paraneoplastic dermatosis, which leads to therapy extension [16]. Most have a specific targeted therapy, although it depends on the malignancy and the severity of the paraneoplastic syndrome. The main treatment and prognosis of the different entities involved are described in Table 3.

**Table 3:** Treatment of paraneoplastic mucocutaneous syndromes in HM. MDS: myelodysplastic syndrome; MGUS: monoclonal gammopathy of uncertain significance; PG: pyoderma gangrenosum; PAN: polyarteritis nodosa; HM: hematologic malignancies; IgA: immunoglobulin A.

Paraneoplastic syndrome	Most common HM	Treatment and prognosis
Neutrophilic dermatoses (Sweet's syndrome)	Acute myeloid leukemia	No specific treatment schemes for malignancy-associated Sweet’s syndrome have been described [13]. Systemic corticosteroids are usually required at a 1 mg/kg dose for three to four weeks [16].
Pyoderma gangrenosum	MDS and MGUS	A systematic review by Montagnon et al. [20] showed that most patients (75%) with HM-associated PG achieved control of the skin lesions with systemic corticosteroids; chemotherapy alone achieved PG resolution in only 7.5% of cases.
Erythema elevatum diutinum	IgA monoclonal gammopathies	First-line treatment is dapsone followed by corticosteroids. Therapy with 50 and 100 mg per day is associated with partial or complete resolution of the disease in most cases. However, a relapse risk exists after therapy ceases in 32% of cases [22-24].
Subcorneal pustular dermatosis	IgA myeloma	First-line treatment is dapsone; other therapies may be used as well such as corticosteroids, sulfasalazine, colchicine, systemic retinoids, phototherapy, and other immunosuppressants such as methotrexate or mycophenolate [25].
Eccrine neutrophilic hidradenitis	Acute myeloid leukemia	Self-resolving entity within days or weeks, without the need for any specific treatment [27]. Topical corticosteroids may be used to reduce the duration of symptoms.
Eosinophilic dermatoses	B-cell chronic lymphocytic leukemia	
Eosinophilic dermatosis associated with hematologic malignancy		No clinical trials are available to guide therapy. Underlying malignancy must be treated. Successful cases have been reported with corticosteroids, antihistamines, phototherapy, doxycycline and nicotinamide, dapsone, and dupilumab [16].
T cell papulosis associated with B-cell neoplasms	B-cell chronic lymphocytic leukemia	The course of this dermatosis is chronic and relapsing, despite treatment [32].
Blistering dermatoses		
Paraneoplastic pemphigus	Hodgkin’s lymphoma	Early directed therapy against malignancy, specifically in the presence of Castleman’s disease or thymoma. First-line therapy is systemic corticosteroids associated with a steroid-sparing agent [16]. In severe or refractory cases, rituximab or intravenous immune globulin may be used [16,33].
Miscellaneous	MDS-associated PAN and hairy cell leukemia	Systemic corticosteroids and HM management have been the cornerstone of management for malignancy-associated vasculitis [35]. This type of vasculitis is characterized by a lower rate of response to corticosteroid/immunosuppressant therapy [16].
Vasculitis		
Dermatomyositis	Non-Hodgkin’s lymphoma	Management is based on immunomodulation with corticosteroids and azathioprine, and malignancy workup for early oncologic treatment [16,42].
Annular granuloma	Lymphomas, specifically Hodgkin’s	Usually generalized and less sensitive to corticosteroid therapy [40,41]. For localized forms, topical steroids. For generalized ones, phototherapy, isotretinoin, dapsone, and hydroxychloroquine.

## Conclusions

Certain hematological neoplasms, such as mycosis fungoides, are generally confined to the skin (and occasionally blood) at diagnosis with no evidence of extracutaneous involvement at the time of presentation. Because of that, it is necessary to know that a heterogeneous group of dermatologic manifestations exists in patients with HM. The presentation may be prior, concurrent, or after the HM diagnosis. Knowing the interaction between neoplastic cells and the immune system is fundamental to developing a specific workup and management for skin diseases associated with HM.
